# Training community mental health staff in Guangzhou, China: evaluation of the effect of a new training model

**DOI:** 10.1186/s12888-015-0660-1

**Published:** 2015-10-26

**Authors:** Jie Li, Juan Li, Graham Thornicroft, Hui Yang, Wen Chen, Yuanguang Huang

**Affiliations:** Guangzhou Brain Hospital, Guangzhou Medical University, 36# Mingxin Road, Liwan, Guangzhou, 510370 China; Henan Provincial Mental Hospital, The Second Affiliated Hospital of Xinxiang Medical University, 388# Jianshe Road, Muye, Xinxiang, 453003 China; Health Service and Population Research Department, Institute of Psychiatry, Psychology and Neuroscience, King’s College London, London, SE5 8AF UK; Faculty of Medical Statistics and Epidemiology, School of Public Health, Sun Yat-sen University,Sun Yat-sen Center for Migrant Health Policy, 74#Zhongshan Road II, Yuexiu, Guangzhou, 510080 China

**Keywords:** Training program, Mental health staff, Stigma, Knowledge

## Abstract

**Background:**

Increasing numbers of people with mental disorders receive services at primary care in China. The aims of this study are to evaluate impact of a new training course and supervision for community mental health staff to enhance their levels of mental health knowledge and to reduce their stigmatization toward people with mental illness.

**Methods:**

A total of 77 community mental health staff from eight regions in Guangzhou in China were recruited for the study.4 regions were randomly allocated to the new training model group, and 4 to the old training model group. Levels of mental health knowledge were measured by purpose-made assessment schedule and by the Mental Health Knowledge Schedule (MAKS). Stigma was evaluated by the Mental Illness: Clinicians’ Attitudes Scale (MICA) and the Reported and Intended Behavior Scale (RIBS). Evaluation questionnaires were given at the beginning of course, at the end, and at 6 month and at 12 month follow-up.

**Results:**

After the training period, the 6-month, and the 12-month, knowledge scores of the intervention group were higher than the control group. At 6-month and 12-month follow-up, means scores of MAKS of the intervention group increased more than the control group (both *p* < 0.05) when age, sex, marriage status, title and time were controlled for. At 6-month follow-up, means scores of MICA of the intervention group decreased more than that of the control group (*p* < 0.01). At after-training, at 6-months, and at 12-months, mean scores of RIBS of the intervention group increased more than the control (*p* < 0.01, *p* < 0.001, *p* < 0.001) when age, sex, marriage status, title and time were controlled for.

**Conclusions:**

Compared with the traditional training course and supervision, the new course improved community mental health staff knowledge of mental disorders, improving their attitudes toward people with mental disorder, and increasing their willingness to have contact with people with mental disorder.

## Background

Mental and substance use disorders accounted for 7.4 % of all disability-adjusted life years (DALYs) worldwide in 2010 [1, 2]. Moreover, the mental health field is facing a serious human resource shortage and a huge treatment gap. Hence, it is a challenge faced by many countries to provide adequate human resources to deliver mental health service to those people who need treatment. This situation more urgently needs to be changed in low-and middle-income countries (LMICs), where 76 to 85 % of people with serious mental disorders have received no treatment in the prior 12 months, whereas this figure is reduced to between 35 to 50 % in high-income countries [3, 4].

To bridge the treatment gap, some researchers have proposed “Task shifting” (also named task sharing) which relies on shifting tasks from specialists to non-specialists to overcome shortages of human resources for mental health [5, 6]. It may be acceptable and feasible to train non-specialists health workers to deliver mental health services in the LMICs [7]. Previous studies have demonstrated that mental disorders can be successfully treated in primary care. Currently experiences suggest that providing assistance and supervision in training primary health care staff to identify and treat people with mental disorders by available specialist mental health staff can promote mental heath service to the public [8, 9].

Besides the limited resources, stigma may be another main factor that hinders people with mental disorder from being treated. Stigma and discrimination are widely experienced by people with mental disorders, even in health-care faculties [10–14]. For this reason, it is not enough to promote the mental heath staff’s knowledge of mental illness. It is more important to train them to combat their own tendencies to stigmatize.

As the provincial capital of Guangdong Province, Guangzhou’s community mental health services have a history of more than half a century. To improve the mental health service status in Guangzhou, Guangzhou Brain Hospital has been in charge of training community mental health staff for a decade and held more than 10 training courses [15]. However, for the community mental health staff, the traditional training curriculum was based on an individual approach, while the public heath approach was lacking. Thus we could not develop effective community mental health services in real situations. Among them, from public health’s point of view, the clinical approach could not offer appropriate training courses for community mental health staff [16]. In order to better deliver mental health services, we are now developing the “Guangzhou model” in the field of community mental health (which also named “PTSA”: Policy, Training, Services and Assessment).

### Aims

Therefore, we introduced a trial in Guangzhou, China, to improve the mental health knowledge of community mental health staff and decrease their stigma related to mental health. Our primary hypothesis is that the new training courses and supervision will significantly improve the mental health staff knowledge. Our secondary hypothesis is that the new training curriculum will decrease the mental health staff stigma and discrimination.

## Methods

### Study design

The study was conducted at the Guangzhou Brain Hospital (also named Guangzhou Psychiatric Hospital), China. The 12 administrative regions in Guangzhou were divided into group of central regions (6 of 12 districts) and group of suburb regions (6 of 12 districts) according to their geographical location. Then, four districts were chosen randomly from each group (4 central districts and 4 suburban districts to constitute the total sample). Next, we randomly allocated 2 districts from each group to constitute the intervention group (2 central districts and 2 suburban districts to constitute the new model group), the other 4 districts named group B (another 2 central districts and 2 suburban districts to constitute the control group). Therefore there were 8 regions involved in the study. The sample size was calculated by the formula:

$$ n=\frac{\left({q}_1^{-1}+{q}_2^{-1}\right){\left({Z}_{\alpha /2}+{Z}_{\beta}\right)}^2{S}^2}{\delta^2} $$

Considering of the attrition (10 %), finally, we got the sample size for the new and traditional model group will be 44, since both groups include four districts. Therefore, the total sample size for the current study was 88.

Then we invited the community mental health staff who worked in the selected districts to participate in the voluntary training. They were told that because of the limited resources, they were trained in two separate periods. All of them were informed that they could leave the training at any time. At last, there were only 40 participants enrolled in the new training model group and 37 participants in the traditional model group.

**Fig. 1:**
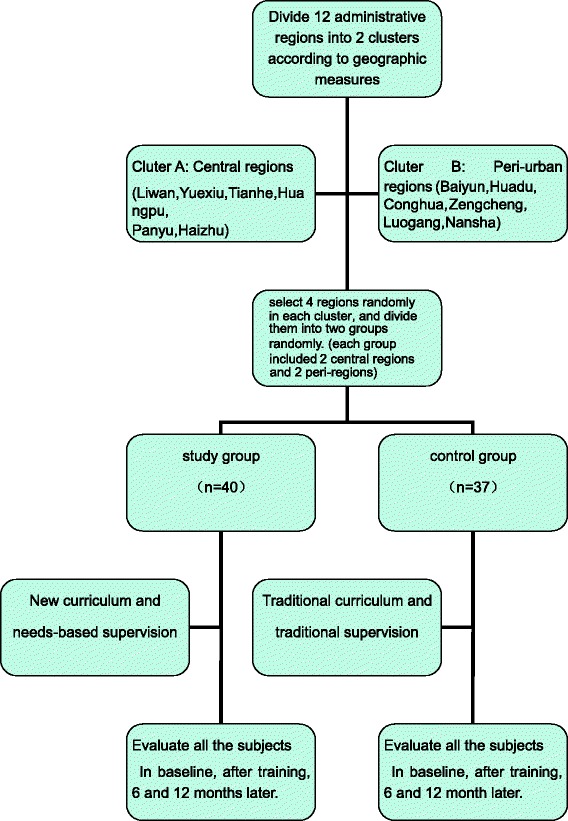
The study flow chart

We trained each group for 14 days respectively, the new curriculum for the new model group, while the old one for the traditional model group. In the new curriculum training, we combine the public and clinical knowledge to set curriculum which used the WHO mhGAP Intervention Guide and the Chinese Medical Association’s guidelines (for the prevention and treatment of schizophrenia and bipolar disorder, etc.) and used a needs-based approach in supervision; while the traditional courses and supervision were used for the traditional model group [17–19]. After 6 months and 12 months, we evaluated the subjects, and compared the results with baseline survey data. Details are shown in Fig. 1.

### Training program

Curriculum Setting: the new curriculum had three modules. The first module mainly included traditional courses, and the second module was clinical practice, and third module combined the public health perspective, stigma and discrimination, adding WHO guidelines, ICD-10, present policies. Traditional courses used the first and second module, which were mainly clinical psychiatric textbooks and clinical practices as usual. Detail are shown in Table 1.

**Table 1:** Curriculum setting

Contents	New curriculum (hours)	Traditional curriculum (hours)
Community mental health	30 (35.3%)	15 (17.6%)
Clinical psychiatry	35 (41.2%)	50 (58.9%)
Clinical practice	17 (20.0%)	17 (20.0%)
Exam	3 (3.5%)	3 (3.5%)
Total (hours)	85	85

Supervision Content: The traditional supervision contents included:

1. Advisory and coordination mechanism, work system, and work flow;
2. To check the quality and quantity of the completion rates of serious mental disorders;

To supervise the personnel and their responsibilities;

(3) To coordinate the funds and allocation;
(4) Task management data and information report of serious mental disorders;
(5) To summarize work performance and advanced examples;
(6) To coordinate, guide and help to solve management and technical problems in the work.

The new supervision is based on the existing traditional supervision, with the following additions:

1. To emphasize the problems in daily work;
2. To strengthen communication with administrators;
3. To coordinate and solve the target problems.

The supervisors mainly consisted of the community psychiatric professionals of Guangzhou Brain Hospital. The matching supervision was provided every three month to each group after the training.

### Sample selection

Inclusion criteria: (1) Mental health staff of community mental health institutions in the target districts of Guangzhou; (2) Graduated above technical secondary school level; (3) Gave informed consent and agreed to accept training and supervising. Exclusion criteria: (1) Failure to complete 80 % of the total courses; (2) Failing to accept follow-up supervision two times or more.

### Measures

We assessed knowledge of mental health using a purpose-made assessment schedule, which contains one part of single choice question, two parts of multiple choice questions and an essay question selected from the textbook named: psychiatric learning guidance and problem sets [20]. Besides, there was another essay question related to public health. The paper consists of 100 score, higher score indicates more knowledge of mental health.

Stigma and discrimination towards mental illness were assessed with three tools: 1) the Mental Health Knowledge Schedule (MAKS). Its total score (part A) was calculated so that higher MAKS score indicates greater stigma-related mental health knowledge. 2) the Mental illness: Clinicians’ Attitudes (MICA) to assess stigmatising attitudes, higher MICA score indicates more negative stigma-related mental health attitudes. 3) the Reported and Intended Behaviour Scale (RIBS): to assess mental health-related reported and intended behavioural discrimination, higher RIBS score indicates greater willingness to contact people with mental illness. These tools of assessment had good psychometric properties [21–26], and our previous research also found that MICA and RIBS had good reliability and validity in China. The internal consistency of the MICA and RIBS were *Cronbach’α* = 0.72, 0.82, respectively [27–29].

### Statistical methods

Analyses of outcomes were based on the intention to treat principle. The Linear Mixed Model was used to show effectiveness of new training intervention and adjust for doctor- level (level-1) potential confounding variables and intra-class correlation (ICC) resulting from clusters (districts) and repeated measures. Unadjusted and adjusted regression coefficients of scores of the theory test with 95 % confidence intervals (CI) were calculated. For the Linear Mixed Model, variance components were chosen as covariance structure for the repeated measures, based on Akaike’s information criteria (AIC). Significance was set at *P* < 0.05. Linear Mixed Model was performed using IBM SPSS Statistics 20.0 (IBM Corporation, USA).

The study was conducted from August 2013 to October 2014. Ethics approval was obtained from Research Ethics Committee of Guangzhou Brain Hospital (Number 66, 2013).

## Results

### Participant characteristics

Seventy-seven community mental health staff gave informed consent to participate. The characteristics of these participants are shown in Table 2. At baseline, there were no significant differences between the two groups in age, education years, sex, marital status except occupation. After a year, 61 (79.2 %) the staff continued to engage in community mental health services, which means that the turnover rate of community mental health staff was 20.8 %. There were no significant differences between the two group (*x*^*2*^ = 0.01, *p* > 0.05).

**Table 2:** Demographic characteristics

Characteristics	New model group (N=40)	Traditional model group (N=37)
Age (years) (χ±SD)	31.83 (6.60)	32.84 (7.06)
Education years (χ±SD)	15.80 (1.56)	16.22 (0.92)
Sex *n* (%)		
Male	16 (47.1)	18 (52.9)
Female	24 (55.8)	19 (44.2)
Marital status *n* (%)		
Single	19 (47.50)	12 (32.43)
Married	21 (52.50)	25 (67.57)
Occupation *n* (%)*		
Doctors	24 (60.00)	32 (86.49)
Sanitarian	13 (32.50)	4 (10.81)
Nurse	3 (7.50)	1 (2.70)

*P < 0.05

### Knowledge of mental health scores

**Fig. 2:**
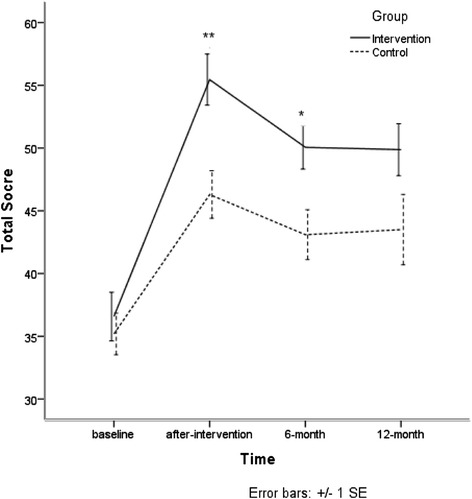
Knowledge of mental health scores

At baseline, knowledge of mental health scores of the intervention group and the control group showed no significant differences. After training, 6-month, and 12-month, knowledge scores of the intervention group were higher than the control group. Especially, the second and third assessments showed statistical significance when age, sex, marriage status, title and time were controlled. At the same time, there is not a significant interaction between group and time. Details are shown in Fig. 2 and Table 3.

**Table 3:** Knowledge of metal health scores

Group	Baseline	After-training	6-month	12-month	*b* (95%CI)	*b*_ad_ (95%CI)^a^
New	36.58 (12.26)	55.45 (12.89)	50.06 (10.12)	49.87 (11.52)	5.94 (1.84–10.05)**	5.75 (1.58–9.93)**
Traditional	35.19 (10.12)	46.30 (11.54)	43.09 (11.22)	43.50 (14.85)		

Intra-class correlation coefficient = 25.86 %

**P < 0.01

^a^Age, sex, education, marriage status, title and time were controlled. There is not a significant interaction between group and time

### Stigma scores

**Fig. 3:**
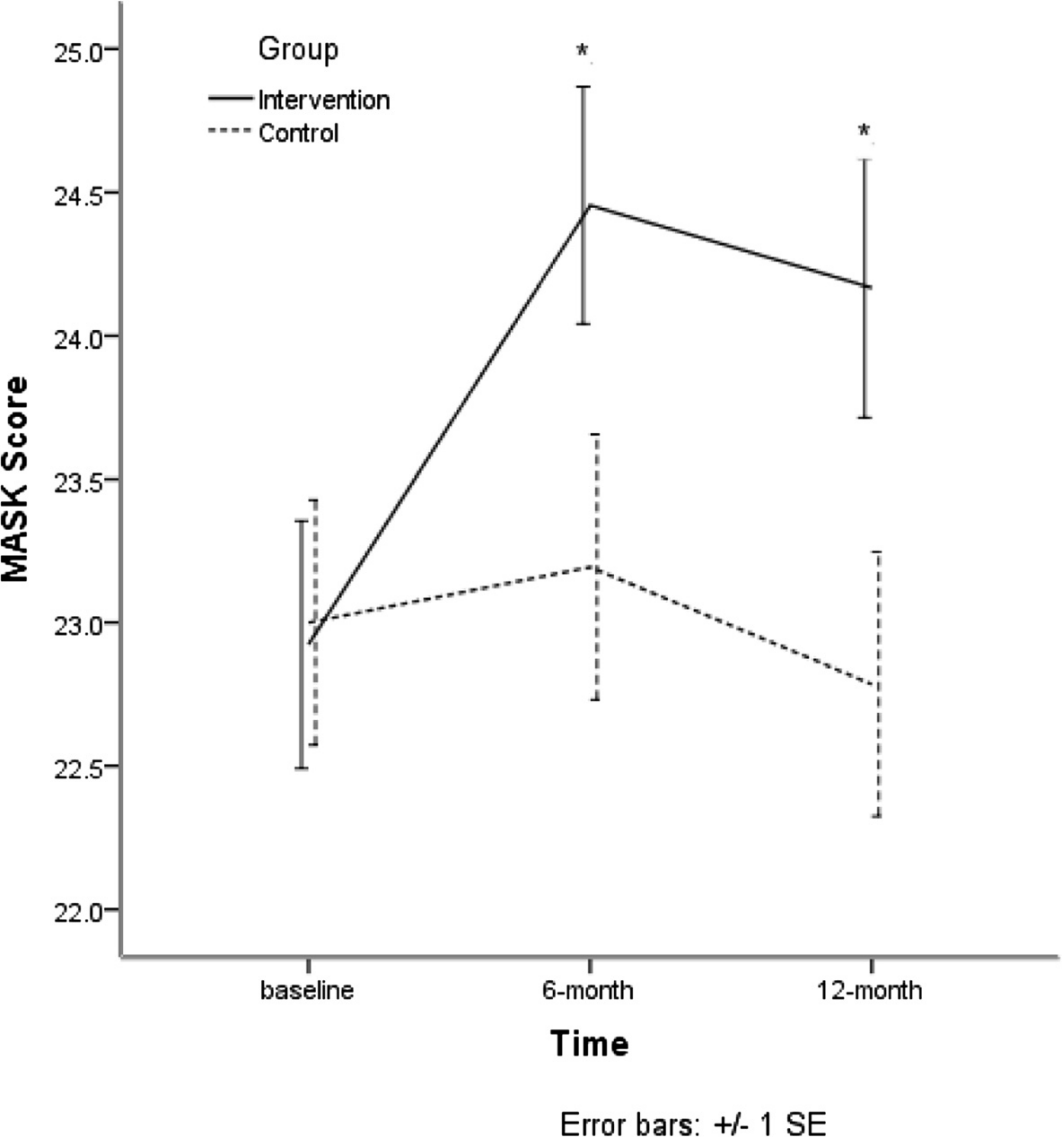
Means scores of MAKS

At baseline, means scores of MAKS, MICA, and RIBS of intervention group and control group showed no significant differences. At 6-month and 12-month, means scores of MAKS of the intervention group increased more than the control group (both *p* < 0.05) when age, sex, marriage status, title and time were controlled . At the same time, there was no significant interaction between group and time. Details are shown in Fig. 3 and Table 4.

**Table 4:** Means scores of MAKS

Group	Baseline	6-month	12-month	*b* (95%CI)	*b*_ad_ (95%CI)^a^
New	22.92 (2.70)	24.45 (2.37)	24.17 (2.48)	1.37 (0.10–2.64)*	1.40 (0.14–2.67)*
Traditional	23.00 (2.56)	23.19 (2.57)	22.79 (2.44)		

Intra-class Correlation Coefficient = 37.11 %

*P < 0.05

^a^Age, sex, education, marriage status, title and time were controlled. There is not a significant interaction between group and time

**Fig. 4:**
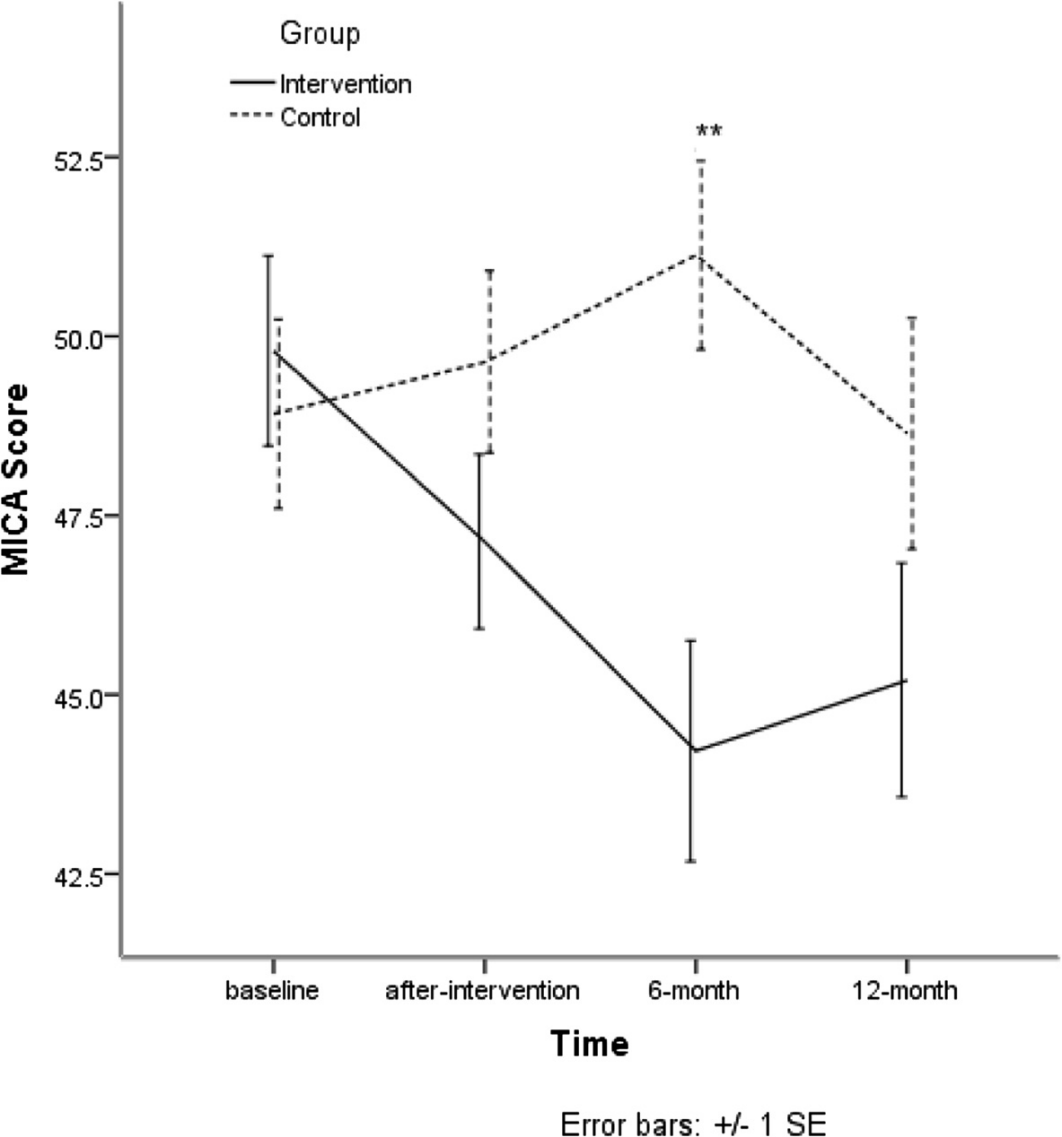
Means scores of MICA

At 6-month, means scores of MICA of the intervention group decreased more than that of the control group (*p* < 0.01). There is a significant interaction between group and time (*p* < 0.001). Details are shown in Fig. 4 and Table 5.

**Table 5:** Means scores of MICA

Group	Baseline	After-training	6-month	12-month	*b* (95%CI)	*b*_ad_ (95%CI)^a^
New	49.79 (8.29)	47.13 (7.50)	44.21 (8.83)	45.20 (8.95)	−3.33 (−7.60-0.95)	−2.21 (−6.42-1.98)
Traditional	48.92 (7.92)	49.64 (7.61)	51.13 (7.32)	48.64 (8.53)		

Intra-class Correlation Coefficient = 28.65 %

^a^Age, sex, education, marriage status, title, time and interaction between group and time were controlled. There is a significant interaction between group and time (*P* < 0.001)

**Fig. 5:**
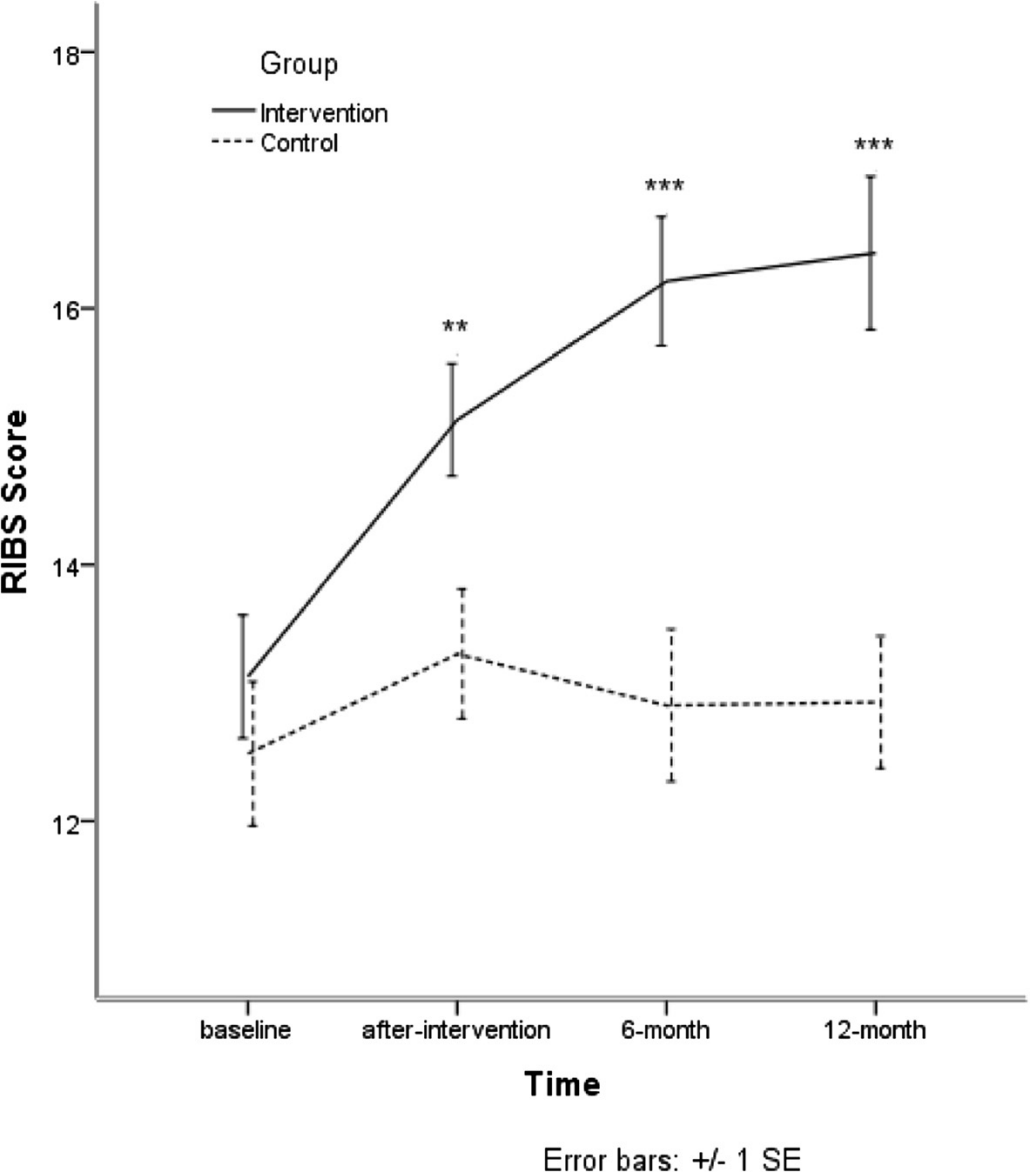
Means scores of RIBS

At after-training, 6-month, and 12-month, mean scores of RIBS of the intervention group increased more than the control group (*p* < 0.01, *p* < 0.001, *p* < 0.001) when age, sex, marriage status, title and time were controlled. There is a significant interaction between group and time.Details are shown in Fig. 5 and Table 6.

**Table 6:** Means scores of RIBS

Group	Baseline	After-training	6-month	12-month	*b* (95%CI)	*b*_ad_ (95%CI)^a^
New	13.13 (3.01)	15.13 (2.71)	16.21 (2.89)	16.43 (3.28)	3.33 (1.81-4.85)***	3.12 (1.75-4.49)***
Traditional	12.53 (3.38)	13.31 (3.04)	12.90 (3.31)	12.93 (2.73)		

Intra-class Correlation Coefficient = 36.07 %

***P < 0.001

^a^Age, sex, education, marriage status, title, time and interaction between group and time were controlled. There is a significant interaction between group and time (*P* < 0.001)

## Discussion

This is the first study, to our knowledge, to follow-up the training effect among community mental health staff in China. Little is known about effective means to reduce stigma of mental health staff in China. At the baseline, there were no significant difference between the two groups in relation to mental health knowledge, attitude and intended behavior to mental disorder. However,after the respective training, the new model group had better knowledge of mental health than the traditional model group,less stigmatizing attitudes, and were more willing to contact people with mental disorder. The results are congruous with other related reports [7, 30, 31]. Moreover,owing to the new model of supervision, these advantages exist over time.

The traditional curriculum settings emphasize clinical psychiatry. However, in the current situation, the community mental health staff were encouraged to manage people with mental disorder, provide health education, rehabilitation training, follow-up, rather than just providing diagnosis and treatment. Notably, merely a broad categories disease, mental disorder is a public health problem. It means that mental health staff should also being equipped with knowledge of community psychiatry, so that they can see problem from a public perspective, and understand the whole range of needs of each patient. In considering these, we modify the structure of the new curriculum to deliver better mental heath service to fully address unmet needs.

Our results indicated that the new curriculum settings also were effective in bringing about a change of attitude toward people with mental disorder. Mental health staff who received the new training model had a more positive attitude toward people with mental disorder and more willing to contact with them. This may make their work more interesting and decrease the human resource flowing in the field of mental health, but we found that there was no significant difference between the new model group and traditional model group, which may reveal a complicated reason that contains the policy and plan of health systems. In the context of seriously shortage of human resource in the field of mental health, stigma related to mental health may be one reason contributing to such a shortage. So, in order to have a stable team, reducing stigma among mental health staff is necessary and is helpful to the delivery of mental health [6, 7, 32–34]. On the other hand, their attitudes toward people with mental disorder may have substantial importance for the patients,such as treatment adherence, recovery, social function [35–37]. In addition, as mental health staff can also be active agent to reduce stigma in others, so it is important that they also have support to reduce their own stigma. [14, 38–41].

It is need to be discussed that there was another study proceeded in this study [28]. There were 40 persons overlapped (all from the new model group) in two studies. Overall, participants in these two studies were overlapped in part, but the study was just a training supervision for the new model group of this study. Therefore, it will not lead to obvious impact on current study. The two studies should be as independent studies.

Our study had some limitations. First, owing to the very limited human resource, only part of the community mental health staff in Guangzhou could participate in the training. So the sample size was relatively small. Second, these trainings’ overall effectiveness is reduced by workload overburden among mental health staff in real-life conditions. Thirdly, we have not assessed whether the training results have an impact on the patients of each group and we will undertake further research to assess effect of training community mental health staff in Guangzhou.

## Conclusions

Our results showed that the training program from a public health perspective and using a needs-based approach in supervision for task shifting is acceptable and feasible and could more decrease community mental health staff’s stigma and discrimination towards patients with mental illness. The change of attitude and behavior is as important as the achievement of knowledge for the delivery of better mental health.
